# Artificial Marker and MEMS IMU-Based Pose Estimation Method to Meet Multirotor UAV Landing Requirements

**DOI:** 10.3390/s19245428

**Published:** 2019-12-09

**Authors:** Yibin Wu, Xiaoji Niu, Junwei Du, Le Chang, Hailiang Tang, Hongping Zhang

**Affiliations:** GNSS Research Center, Wuhan University, No. 129 Luoyu Road, Wuhan 430079, China; ybwu@whu.edu.cn (Y.W.); dujunwei@whu.edu.cn (J.D.); changlesgg@whu.edu.cn (L.C.); thl@whu.edu.cn (H.T.); hpzhang@whu.edu.cn (H.Z.)

**Keywords:** multirotor UAV, precision landing, artificial marker, pose estimation, sensor fusion

## Abstract

The fully autonomous operation of multirotor unmanned air vehicles (UAVs) in many applications requires support of precision landing. Onboard camera and fiducial marker have been widely used for this critical phase due to its low cost and high effectiveness. This paper proposes a six-degrees-of-freedom (DoF) pose estimation solution for UAV landing based on an artificial marker and a micro-electromechanical system (MEMS) inertial measurement unit (IMU). The position and orientation of the landing maker are measured in advance. The absolute position and heading of the UAV are estimated by detecting the marker and extracting corner points with the onboard monocular camera. To achieve continuous and reliable positioning when the marker is occasionally shadowed, IMU data is fused by an extended Kalman filter (EKF). The error terms of the IMU sensor are modeled and estimated. Field experiments show that the positioning accuracy of the proposed system is at centimeter level, and the heading error is less than 0.1 degrees. Comparing to the marker-based approach, the roll and pitch angle errors decreased by 33% and 54% on average. Within five seconds of vision outage, the average drifts of the horizontal and vertical position were 0.41 and 0.09 m, respectively.

## 1. Introduction

With the rapid development of micro-electromechanical systems (MEMSs) and high-performance flight control processors, applications and research of multirotor unmanned air vehicles (UAVs) have received increasing attention worldwide. Due to the unique structure and stability, multirotor UAVs are widely used in precision agriculture [1,2], rescue [3], surveillance [4,5], etc. These applications also pose new challenges for the precision landing of UAVs [6]. To land safely and precisely, the localization of UAV requires high accuracy, continuity, and reliability.

Global navigation satellite system/inertial navigation system (GNSS/INS)-integrated navigation [7] technologies are the most widely used navigation methods for various UAVs [8,9]. However, the precision of the integrated navigation positioning is completely dependent on the GNSS. Although decimeter-level positioning results can be obtained by a consumer-grade dual-frequency GNSS receiver with sufficient satellites [10,11], in areas with low satellite observation such as city canyons and forests, positioning errors will increase. Moreover, it is totally unavailable for indoor applications. To ease these problems, vision-based pose estimation approaches for UAV landing have attracted extensive research due to their low cost and ability to sense the environment [12,13]. A survey of the vision-based landing techniques for UAVs is presented in [14]. In most applications, e.g., precision agriculture, we can determine the landing point of UAVs where we are allowed to place a customized pad in advance to take advantage of the pertaining features. 

Abundant works concerning the design of the fiducial marker system have been proposed in literature, such as ARTag [15], AprilTag [16], and ArUco [17]. The authors in [18,19] use AprilTag markers as their landing pad. In [20], ARTag markers are used for UAV localization. Researchers also have proposed specialized markers to meet precision landing requirements of UAVs.

The authors in [21] use video from the onboard camera and the orientation and altitude of the ground vehicle to calculate the UAV position with respect to (w.r.t.) the target. In [22], an integration algorithm of monocular and stereo vision is exploited to estimate the approach angle and relative height of a UAV by Hough transform and vanishing-line geometry. In [23], the authors introduce a dynamic fiducial marker that can change its appearance according to the requirements of the UAV. A landing marker which consists of the letter “H” inside a black circle is presented in [24]. A landing pad composed of markers of different size is utilized to permit detection from high as well as very low altitude in [25]. In [26], the authors describe a pattern comprising several concentric white rings on a black background. The rings are identified by the unique ratio between their inner and outer border radius. The authors in [27] present a landing marker consisting of three inner circles, each of which is evenly divided into eight fan areas. The area between the inner circles is filled by an even distribution of black and white. They use the template-matching strategy for marker detection in daytime, and a morphology technique named the hit-and-miss algorithm at nighttime. In 2018, the authors extended this work by introducing a method to exploit the trained features extracted from a convolutional neural network (CNN) for marker tracking, which can be performed at very high altitude [28]. The authors in [29] propose a graphics processing unit (GPU)-based pose estimation method based on a marker composed of circles and use experimental covariance measurements to estimate the camera pose during occlusions and vibrations. A comparison of the state of the art on the autonomous landing of UAVs using artificial markers is also shown in this work, regarding the marker shape, positioning error, frame rate, etc. Authors in [27] classify the previous vision-based landing systems for UAVs into two categories (namely passive and active methods) and compare their strength and weakness.

In the above marker-based positioning methods, when the marker is severely occluded, the system will not work. Furthermore, these approaches do not take into consideration the absolute heading angle, although some of them do retrieve the relative heading. To increase the positioning rate as well as cover the short-period marker occlusions, inertial measurements can be introduced to fuse with image data.

The authors in [24] use projective geometry to estimate the five-degrees-of-freedom (DOF) pose from the elliptic projection of the marker. Then the gravity vector estimated by the inertial measurement unit (IMU) is incorporated to resolve the remaining geometric ambiguity. In [25], the authors perform a homography-based method to obtain UAV pose from the marker and integrate it with the IMU data in a loosely coupled way. Their work focus on the landing of UAVs on a moving platform. However, the horizontal angles, which are essential for flight control, are not estimated in the system. Moreover, the IMU sensor errors are not modeled, which can result in significant position error accumulation with time.

In this work, a six-DoF state estimation system based on an artificial marker, a visible light monocular camera, and a MEMS IMU to meet the requirements of multirotor UAV landing is introduced. The IMU used in our system is at dollar-level, which is widely used for commercial smartphones. Referring to the prior works about marker design and detection systems, an improved methodology applicable to UAV landing is proposed. The inner corners of the marker are used for pose estimation when the UAV is too close to the ground and the whole marker is out of the image frame. IMU data is fused by an extended Kalman filter (EKF) to increase the navigation result rate to permit aggressive maneuvers and to cope with short-time marker occlusion. This also results in more smoothing results for both position and attitude. Unlike previous visual/inertial fusion system for UAV landing, models of the error components of IMU are considered and roll and pitch angles are also predicted by INS and updated in the EKF. Field tests illustrate the magnificent performance of our system. It worth mentioning that this work only focuses on the pose estimation for the UAV landing instead of on the whole closed-loop control system.

This paper is organized as follows. In Section 2, the design and detection of the marker as well as the relative state estimation method with the extracted marker is presented. Then the fusion algorithm of pose information from the marker and IMU data is described. Experimental evaluations of the proposed system are provided and discussed in Section 3. Finally, Section 4 draws some conclusions.

## 2. Methods

### 2.1. Marker Detection

Similar to previous works, the marker proposed in this work is a big square consisting of small black and white squares. They are distributed on a plane according to a certain rule with the advantages of large information capacity and good expandability. 

The marker is constituted by N×N black and white squares of the same size. The number and size of the squares are dependent on the size of the dataset, as well as the amount of encoded information. The border of the marker is black with several black squares properly filled inside. To avoid the central symmetry of the marker, which leads to ambiguous heading definition, it must be ensured that the ultimate marker is not centrosymmetric when determining internal black squares. Our marker is specifically designed for UAV landing, reducing the computational cost while limiting false positives. Unlike the aforementioned markers, the structure inside the border of the proposed marker is much simple. To meet the requirement of estimating pose at different heights along the landing path especially when the UAV is close to the ground, a black square is set at the marker center. At the beginning, the UAV detects the whole marker and adjust its horizontal position and heading while landing. Once it descends to a certain height where the marker is too large to be detected, the center square can be used for guidance. Thus, N should be an odd number.

**Figure 1:**
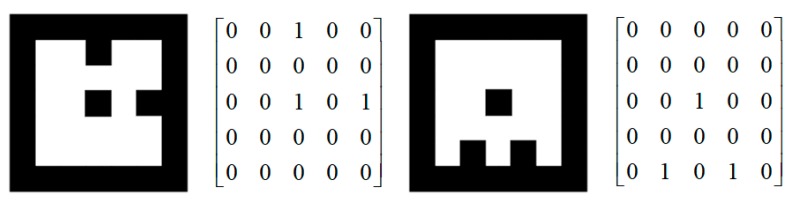
Examples of the proposed marker and binary encoding matrix.

A marker database is consequently built for different landing points. Every marker has its own specific number and involves a different command for the UAV. We use a binary bit stream to represent the coding matrix of the marker for fast and accurate matching, with values of 0 for black and 1 for white. The (N−2)×(N−2) matrix uniquely represents the encoding information of the marker. Figure 1 shows examples of two different markers (N=7) and their encoding matrices.

**Figure 2:**
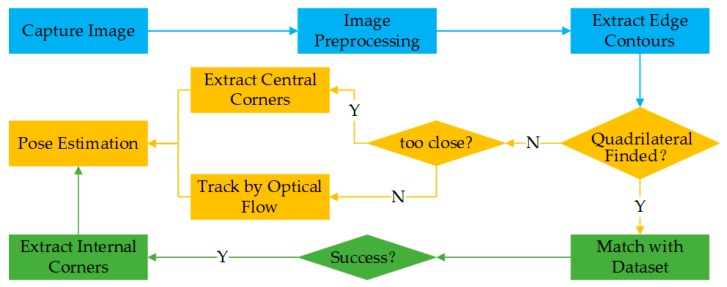
Flow chart of pose estimation by detecting the marker.

**Figure 3:**
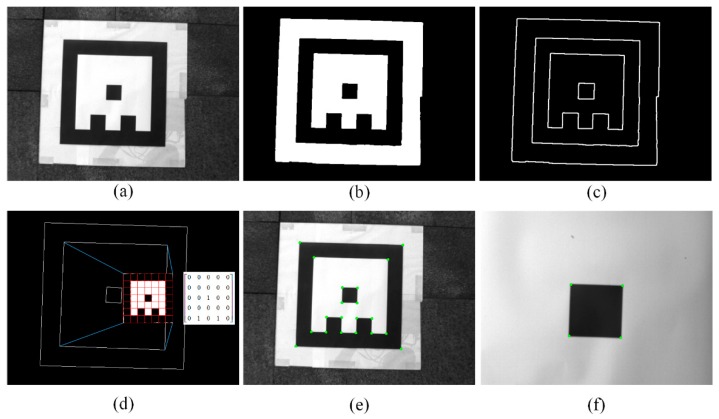
Image processing results. (**a**) Original image; (**b**) binary image after Otsu binarization; (**c**) polygon contours extracted in (**b**); (**d**) quadrilateral contours and the matched marker; (**e**) the corner points extracted on the marker; (**f**) the extracted corner points of the central marker when the unmanned air vehicle (UAV) is very close to the ground.

The main process of pose estimation by the marker is shown in Figure 2. First, we perform histogram equalization and morphological processing on the captured image to reduce the noise and then execute binarization. The Otsu [30] algorithm is employed since it provides the optimal image threshold given that the pixel distribution is bimodal, which is true in this scenario. We use the Canny [31] operator to extract the contours in the image and the Douglas–Peucker algorithm to fit the contours to a polygon. After removing the unreasonable contours, all the quadrilateral contours are extracted, and the internal pixels are extracted and clustered to match the encoding matrix in the database. After determining the unique matrix, the vertices of the marker on the image are obtained, and all corners inside the marker are extracted [32]. If the entire marker cannot be extracted, the corners are tracked by calculating the optical flow [33]. When the UAV is too close to the marker, the corners of the central black square are extracted. Since the positions of the corner points are known, the position and orientation of the camera can be estimated by a set of correspondences between real world points and their projection on image. This is introduced in Section 2.2. Figure 3a–f presents the image processing results of the proposed system.

### 2.2. Pose Estimation by Marker

**Figure 4:**
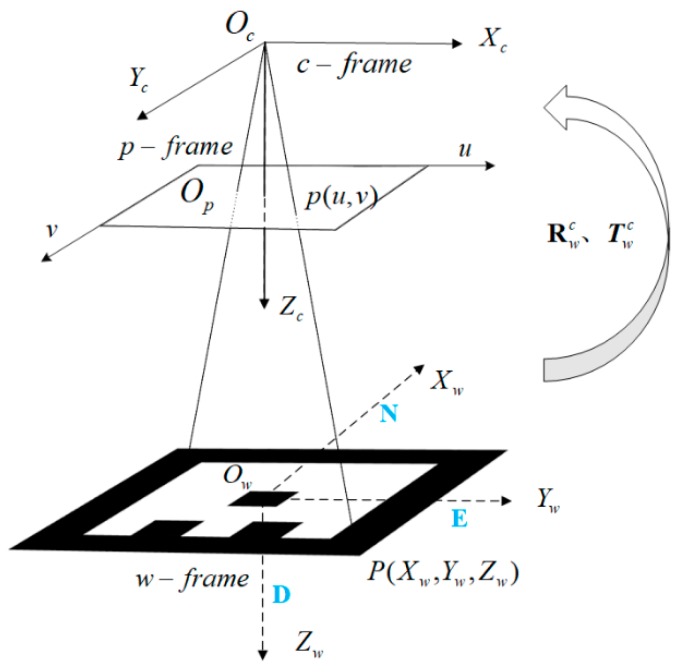
Definition of coordinate systems and camera projection model. $O_{c}$ is the optic center of the camera and $O_{c}-X_{c}Y_{c}Z_{c}$ denotes the c-frame; $O_{p}-uv$ denotes the p-frame; and $O_{w}-X_{w}Y_{w}Z_{w}$ denotes the w-frame.

The relative pose between two coordinate systems can be retrieved by matching the corresponding points. The world coordinate system (w-frame) is established with the center of the marker as the origin. The camera coordinate system (c-frame) is established with the optical center of the camera as the origin, *x*-axis pointing to the right, *y*-axis pointing down. The pixel coordinate system (p-frame) is established with the upper left corner of the image as the origin and (u,v) parallel to (x,y) of the c-frame, respectively. Figure 4 shows the coordinate systems. The camera projection model is shown as Equation (1). The marker can be laid arbitrarily at the landing point, but we have to measure its heading angle in advance for UAV absolute heading estimation. For easy understanding, we assume that the *x*-axis and *y*-axis of the w-frame points to the north and east, respectively.

The transformation among coordinate systems is given as

(1) $$P^{c}=R_{w}^{c}P^{w}+T_{w}^{c},$$

(2) $$Z_{c}\left[\begin{array}{c} u\\ v\\ 1 \end{array}\right]=\left[\begin{array}{ccc} f_{x} & 0 & c_{x}\\ 0 & f_{y} & c_{y}\\ 0 & 0 & 1 \end{array}\right]\left[\begin{array}{c} X_{c}\\ Y_{c}\\ Z_{c} \end{array}\right]=KP^{c},$$

where $P^{c}$ and $P^{w}$ are the coordinates of the corner points in c-frame and w-frame, respectively; (u,v) are the coordinates of the corner points in p-frame; $R_{w}^{c}$ and $T_{w}^{c}$ are the rotation matrix and translation vector from w-frame to c-frame, respectively; K is the intrinsic parameter matrix of the camera, which can be calibrated by the chessboard method proposed in [34]; $\left(c_{x},c_{y}\right)$ is the camera principal point in p-frame; and $f_{x},f_{y}$ represent the focal length of the camera in terms of pixel dimensions in the x and y direction, respectively. According to the matching pairs of the corners in w-frame and p-frame, the estimation of the UAV pose $\left(R_{w}^{c},T_{w}^{c}\right)$ is a perspective-n-point (PNP) problem that can be solved by [35]. The random sample consensus (RANSAC) method [36] is introduced to exclude corner outliers to ensure the robustness of the visual positioning. Then, we regard it as the initial value and perform the Levenberg–Marquardt [37] approach to optimize it according to the minimum reprojection error criterion, shown as

(3) $$\underset{R_{w}^{c},T_{w}^{c}}{\min}\sum_{i\in n}\rho_{i}||P_{i}-\frac{1}{Z_{c}}K(R_{w}^{c}P^{w}+T_{w}^{c})||,$$

where i∈n denotes the ith point of the all n points; $\rho_{i}$ indicates the weight factor. To reduce the influence of a corner observation with a large error on the result, we use the Cauchy loss function (Equation (4)) to reduce the weight of this observation [38].

(4) ρ(s)=log(1+s)

The relative position of the camera w.r.t. the w-frame can be transformed to the absolute pose in the e-frame by the following equations,

(5) $$\delta r^{e}=D\delta r^{n},$$

(6) $$D=\left[\begin{array}{ccc} R_{M}+h & 0 & 0\\ 0 & (R_{N}+h)\cos\varphi & 0\\ 0 & 0 & -1 \end{array}\right],$$

where $\delta r^{e}$ is the position vector from the origin of w-frame to the camera center projected in the e-frame; $\delta r^{n}$ is the camera coordinates in the n-frame; $R_{M}$ is the meridian radius of curvature; $R_{N}$ is the radius of curvature in the prime vertical; φ is the geodetic latitude; and h is the altitude.

### 2.3. Visual/Inertial Fusion

**Figure 5:**
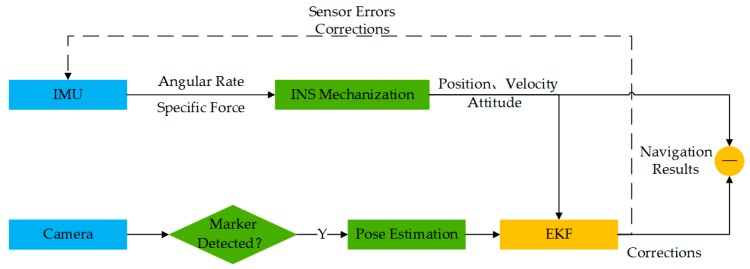
System overview of the camera/INS (inertial navigation system) integrated navigation. The vehicle pose is retrieved by the two sensors independently and then fused by error-state extended Kalman filter (EKF), namely loosely coupled.

INS is able to calculate UAV pose from the initial state by IMU measurements alone. By detecting the marker, the relative position and heading of the UAV in the w-frame is retrieved which provides reliable external observations for INS to correct its errors. Since the corner points are on the same plane that is parallel to the horizontal plane, the observability of the horizontal angle (i.e., roll and pitch angle) is weak for PNP. For INS, the external position assist and gravity constraint make the horizontal angle observable. Moreover, the IMU data rate is higher than that of the camera in general. INS mechanization can predict the UAV state during two consecutive image frames. Due to the magnificent complementarity, it is valuable to fuse the two sensors’ data. The algorithm overview is presented in Figure 5.

#### 2.3.1. INS Mechanization and System Model

The main procedure of the INS mechanization can be divided into three steps:

1. Integrate the angular rate to update the vehicle attitude;
2. Transform the acceleration to the n-frame by the updated attitude, then integrate it to obtain the vehicle velocity;
3. Integrate the velocity to calculate the vehicle position.

Refer to [39] for the detailed update equations.

The demands for an accurate estimation of the vehicle pose necessitate the modeling and online estimation of the error components of the sensor. The state vector of the integrated navigation system constructed in this paper can be expressed as

(7) $$x(t)=\left[\begin{array}{ccc} \delta r^{n} & \delta v^{n} & \begin{array}{ccccc} \phi & b_{g} & b_{a} & s_{g} & s_{a} \end{array} \end{array}\right],$$

where $\delta r^{n}$ is the position error in the navigation frame (n-frame), which has its origin coinciding with that of the b-frame, with its *x*-axis pointing north, *z*-axis pointing down, and *y*-axis completing a right-hand orthogonal frame, i.e., the north–down–east (NED) system; $\delta v^{n}$ is the velocity error in the n-frame; ϕ is the attitude error, composed of roll error, pitch error, and heading error; $b_{g}$ and $b_{a}$ are the residual bias error of the gyroscope and the accelerometer, respectively; $s_{g}$ and $s_{a}$ are the residual scale factor error of the gyroscope and the accelerometer, respectively.

The continuous time system is modeled as,

(8) $$\dot{x}(t)=F(t)x(t)+G(t)w(t),$$

where, F(t) is the system dynamics matrix; G(t) is the noise distribution matrix of the system; and w(t) is the vector describing the system noise.

Due to the uncertainty in the sensors and the gravity field, the navigation parameters obtained from the INS mechanization equation contain errors. The phi-angle error model [40] is used here. Thus, the differential equation of the inertial navigation error is as follows:

(9) $$\dot{\phi }=-\omega_{in}^{n}\times \phi +\delta \omega_{in}^{n}-C_{b}^{n}\delta \omega_{ib}^{b},$$

(10) $$\delta {\dot{v}}^{n}=C_{b}^{n}\delta f^{b}+C_{b}^{n}f^{b}\times \phi +v^{n}\times (2\delta \omega_{ie}^{n}+\delta \omega_{en}^{n})-(2\omega_{ie}^{n}+\omega_{en}^{n})\times \delta v^{n}+\delta g_{l}^{n},$$

(11) $$\delta {\dot{r}}^{n}=-\omega_{en}^{n}\times \delta r^{n}+\delta \theta \times v^{n}+\delta v^{n},$$

(12) $$\delta \theta =\left[\begin{array}{c} \delta r_{E}/\left(R_{N}+h\right)\\ -\delta r_{N}/\left(R_{M}+h\right)\\ -\delta r_{E}\tan\varphi /\left(R_{N}+h\right) \end{array}\right]$$

where, b denotes the body frame (b-frame), which has its axes coinciding with the IMU’s body; $\delta \omega_{ib}^{b}$ and $\delta f^{b}$ are the gyroscope measurement error and the accelerometer measurement error, respectively; $\omega_{ie}^{n}$ is the rotation rate vector of the e-frame with respect to the inertial frame (i-frame) projected to the n-frame; $\omega_{en}^{n}$ is the rotation rate vector of the n-frame with respect to the e-frame projected to the n-frame; $\delta g_{l}^{n}$ is the gravity vector projected to the n-frame; $C_{b}^{n}$ is the rotation matrix from the body frame (b-frame) to the n-frame; $\delta r_{N}$ and $\delta r_{E}$ are the position error on north and east direction of the n-frame, respectively; The variables $b_{g}$, $b_{a}$, $s_{g}$, and $s_{a}$ are modeled by a first-order Gauss–Markov process.

(13) $$\begin{array}{c} \dot{x}=-\frac{1}{T}x+w\\ x_{k+1}=e^{-\Delta t_{k+1}/T}x_{k}+w_{k} \end{array},$$

where T is the correlation time of the process, and w is the driving white noise process.

#### 2.3.2. Linearized Measurement Model

Since the IMU and the camera cannot be installed at the same place with parallel axes, the pose retrieved from the marker is different from that derived from the INS, which is known as the lever-arm effect and installation angle effect, as described in Equations (14) and (15), respectively. These misalignments have to be calibrated in advance or they will cause significant errors in both position and attitude. For easy understanding, it is assumed that the b-frame coincides with the UAV coordinate system (v-frame).

**Figure 6:**
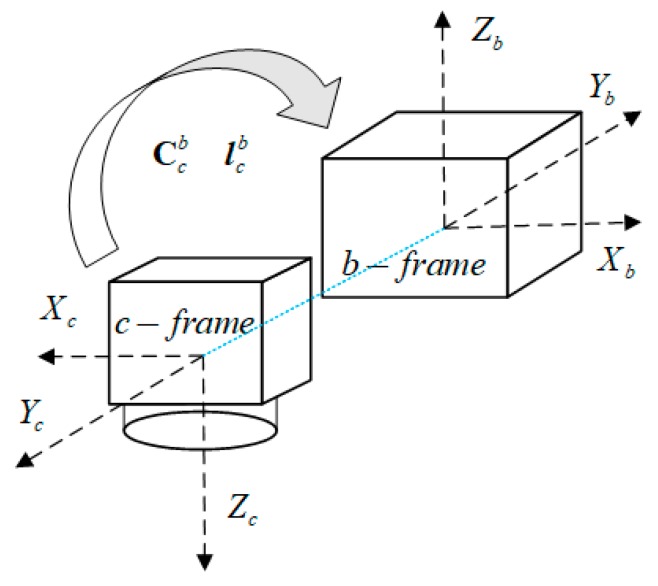
Installation relationship between the camera and inertial measurement unit (IMU).

The installation relationship between the camera and IMU is depicted in Figure 6. 

The camera position derived from the IMU and camera is expressed as follows,

(14) $$\begin{array}{ll} {\hat{r}}_{c}^{e} & ={\hat{r}}_{IMU}^{e}+C_{n}^{e}{\hat{C}}_{b}^{n}l_{c}^{b}\\ & =r_{IMU}^{e}+\delta r_{IMU}^{e}+C_{n}^{e}\left[I-\left(\phi \times \right)\right]C_{b}^{n}l_{c}^{b}\\ & =r_{c}^{e}+\delta r_{IMU}^{e}+C_{n}^{e}\left(C_{b}^{n}l_{c}^{b}\times \right)\phi \\ {\tilde{r}}_{c}^{e} & =r_{c}^{e}+C_{n}^{e}n_{rc} \end{array},$$

where ${\hat{r}}_{c}^{e}$ is the camera position calculated by the IMU-derived position and lever arm; ${\tilde{r}}_{c}^{e}$ is the camera position estimated by marker detection; $l_{c}^{b}$ is the lever arm vector from the IMU center to the camera center in the b-frame; $n_{rc}$ is the position error of visual estimation; and []× represents the antisymmetric matrix of a vector.

Since the landing maker can provide a reliable heading reference, it can also be exploited to correct INS errors as an aiding source. The camera attitude derived from the IMU and camera are expressed as follow,

(15) $$\begin{array}{l} {\hat{C}}_{c}^{n}={\hat{C}}_{b}^{n}C_{c}^{b}=\left[I-\left(\phi \times \right)\right]C_{b}^{n}C_{c}^{b}\\ {\tilde{C}}_{c}^{n}=\left[I+\left(v\times \right)\right]C_{c}^{n} \end{array},$$

(16) $$\begin{array}{c} {\hat{C}}_{c}^{n}=[I-(\rho \times )]{\tilde{C}}_{c}^{n}\\ \left[I-\left(\phi \times \right)\right]=\left[I-\left(\rho \times \right)\right]\left[I+\left(v\times \right)\right] \end{array},$$

(17) $$\begin{array}{c} \rho \times =I-{\hat{C}}_{c}^{n}{\tilde{C}}_{c}^{n}{}^{T}\\ \rho =\phi +v \end{array},$$

where ${\hat{C}}_{c}^{n}$ and ${\tilde{C}}_{c}^{n}$ are the camera attitude matrices derived from the INS and recovered from the visual solution, respectively; $C_{c}^{b}$ is the rotation matrix from the c-frame to the b-frame; v is the heading error of the visual estimation; and ρ is the attitude difference between the vehicle attitude calculated by INS and vision. Hence, the observation vector can be expressed as a combination of the position error observation and heading error observation as follows:

(18) $$Z=\left[\begin{array}{c} z_{rc}^{n}\\ \rho_{heading} \end{array}\right],$$

where, $\rho_{heading}$ is the third element of ρ and $z_{rc}^{n}$ can be written as

(19) $$\begin{array}{ll} z_{rc}^{n} & =C_{e}^{n}\left({\hat{r}}_{c}^{e}-{\tilde{r}}_{c}^{e}\right)\\ & =C_{e}^{n}\left(\delta r_{IMU}^{e}+C_{n}^{e}\left(C_{b}^{n}l_{c}^{b}\times \right)\phi -C_{n}^{e}n_{rc}\right)\\ & =\delta r^{n}+(C_{b}^{n}l_{c}^{b}\times )\phi -n_{rc} \end{array}.$$

## 3. Experimental Results Discussion

### 3.1. Experiments Description

**Figure 7:**
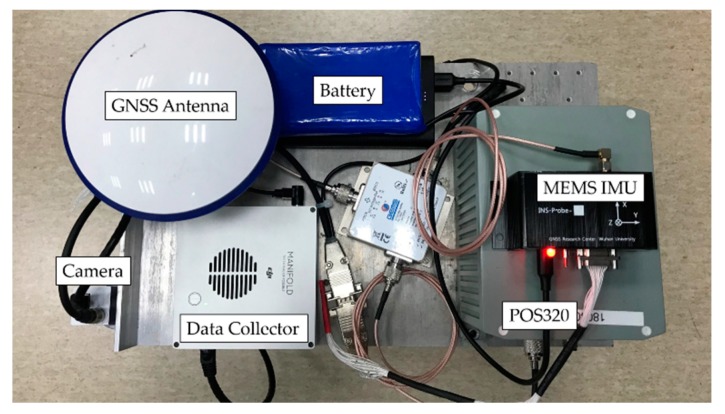
Experimental equipment.

To demonstrate the performance of the proposed system, we carried out field tests and analyzed the results from two aspects: the retrieved pose errors and reprojection errors of the corner points. The GNSS and high-precision fiber-optic IMU (POS320, MAP Space Time Navigation Technology Co., LTD, Wuhan, China) integrated navigation systems were used as references for the pose. The camera and consumer grade MEMS IMU (ICM20602)-integrated navigation system was the system under test. The camera used in the experiments was a global shutter camera with a 644 × 484 resolution (Mako G-030C, Allied Vision, Stadtroda, Germany). The IMU data rate was 50 fps, and the image frame rate was 20 fps. Figure 7 shows the experimental equipment platform. The spatial displacement of the IMU and the camera was calibrated in advance [41]. Table 1 provides the technical parameters of the two IMUs. The position and heading of the marker in the e-frame were previously obtained by GNSS post processed kinematic (PPK) static measurements. The size of the marker used in the tests was 29 cm × 29 cm.

**Figure 8:**
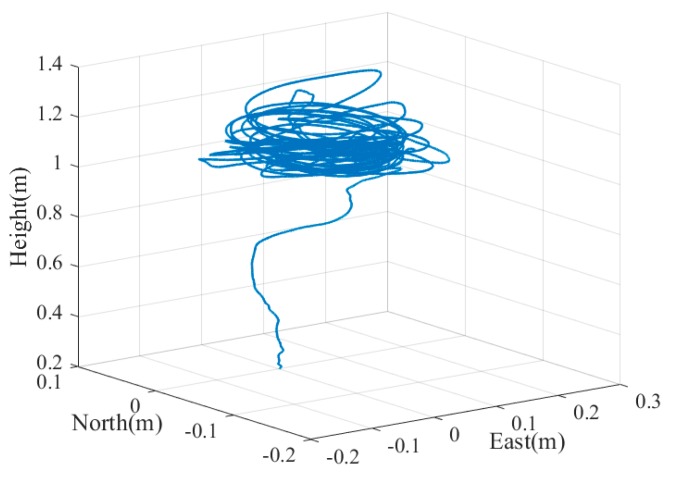
Trajectory of the test platform in test 05.

**Figure 9:**
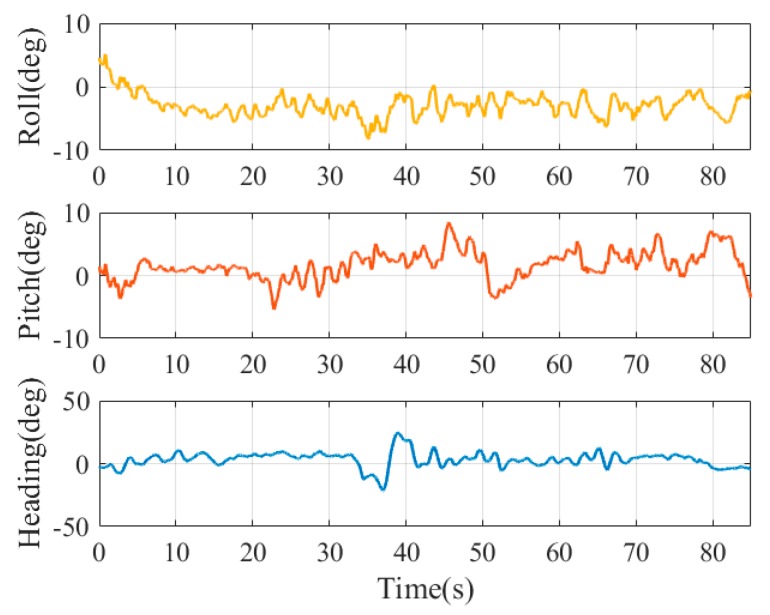
Attitude variation of the test platform in test 05.

**Figure 10:**
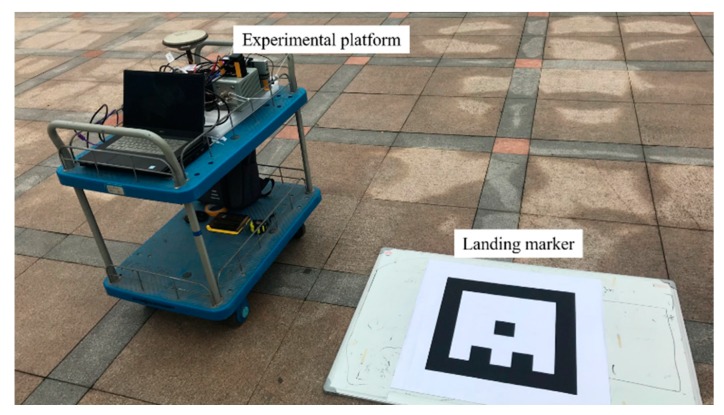
Experimental environment.

Without the expensive motion capture system, it is too difficult, if not impossible, to obtain a reliable vehicle pose as ground truth with light load. A machine with four stepper motors, allowing precise movement of the camera in 3D space, was designed and built in [29] to evaluate the estimated position of the camera w.r.t. the maker. Although the high-precision GNSS/INS integrated systems can provide six-DOF pose references, they are usually too heavy (more than 10 kg) to be mounted on the UAV (like the one used in our experiments). Therefore, we hold the experimental device with reference system by hand to mimic the motion and trajectory of the UAV when landing to evaluate the pose accuracy convincingly. The GNSS/INS pose reference system was fixed on a rigid platform with the camera and MEMS IMU. The position and angle misalignment between the two IMUs was calibrated previously to project the ground truth to the MEMS IMU center. The position of the antenna phase center relative to the reference system was also measured in advance. The camera, IMU, and GNSS data were collected for postprocessing. We performed post processed kinematic (PPK) for the GNSS data process, then the smoothing results of the GNSS/INS integrated navigation were calculated as ground truth. The experiments were executed five times on Friendship Square at Wuhan University on 28 February 2019. The trajectory and attitude variation of the test platform in the fifth test is shown in Figure 8 and Figure 9. The experimental environment is shown in Figure 10.

### 3.2. Results Discussion

**Figure 11:**
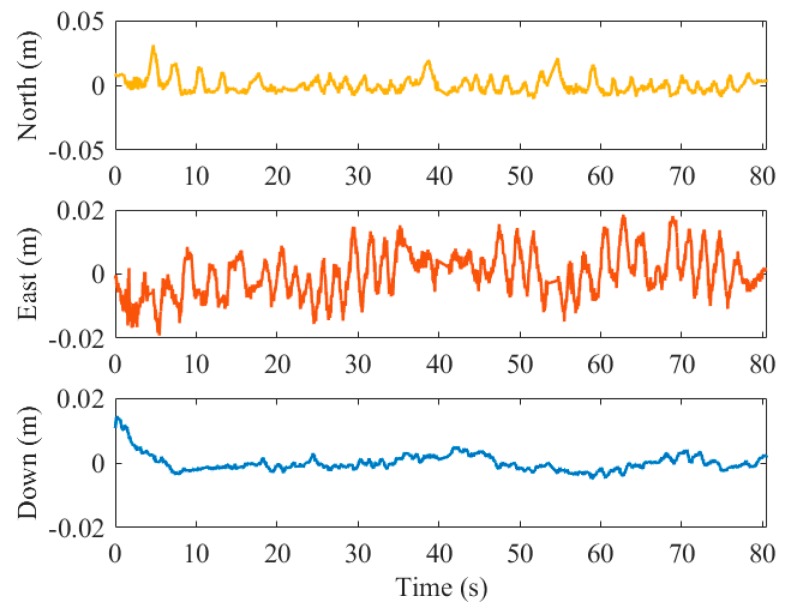
Position errors of the visual solution in test 03.

**Figure 12:**
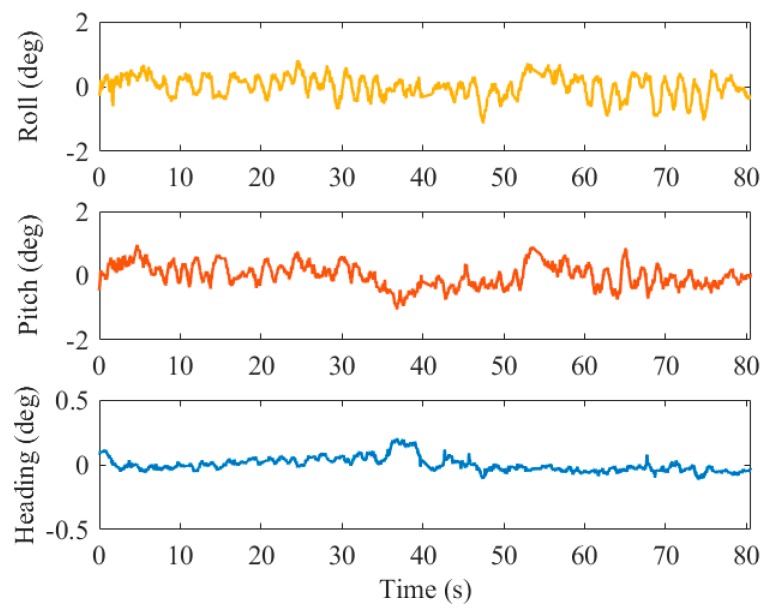
Attitude errors of the visual solution in test 03.

The position and attitude errors of the results by the pure visual solution in the third test are shown in Figure 11 and Figure 12, respectively.

The statistical results of the position and attitude errors of the visual solution for five experiments are shown in Table 2 and Table 3, respectively. The maximum and mean values of the error are calculated by the absolute error sequence over the whole article.

The tables show that the positioning error of the visual solution does not exceed 0.01 m in any of the three directions. It can be observed that the positioning results on horizontal direction are less accurate and more unstable than that on vertical direction. Given that the available satellite number was beyond 20 and the carrier-phase ambiguities were resolved to their integer values in our experiments, the accuracy of PPK smoothing positioning could be thought to be at the centimeter level [42]. Meanwhile, it is obvious that the noise influences of visual positioning and PPK positioning are independent of each other, hence the absolute positioning accuracy of the proposed visual solution is equivalent to that of the smoothed PPK.

For the attitude errors, the root mean square error (RMSE) of the roll angle was 0.45° (1σ), the pitch angle error was 0.43° (1σ), and the heading angle error was 0.1° (1σ). Since the corners of the marker are all on the horizontal plane (almost parallel to the p-frame), we can calculate the heading angle very accurately based on the position of the corresponding points on the image, but the roll and pitch estimations are less stable because of the lack of observations in the vertical direction.

The statistical results of the position and attitude errors of the visual/inertial fusion solution in five experiments are shown in Table 4 and Table 5, respectively.

The statistical results show that the positioning errors of the visual/inertial integrated solution are not more than 0.01 m in any of the three directions, which is consistent with the vision solution.

**Figure 13:**
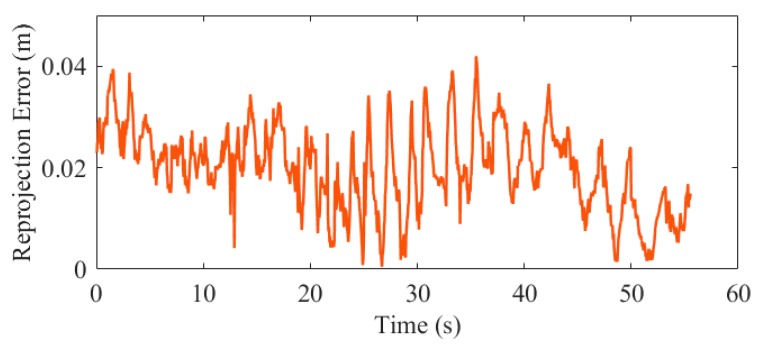
Corner points reprojection errors on the horizontal plane in test 01.

In addition, the reprojection errors was calculated to prove the pose accuracy. The corner points on the image that are not used for estimation (rejected by the RANSAC) were projected into the w-frame by the estimated pose, and the errors comparing to the real 3D corners on the horizontal direction were calculated. The results of the first test are shown in Figure 13, and the statistical results of the five experiments are shown in Table 6.

The statistical results show that the corner reprojection error is 0.022 m (1σ), which also verifies that the position accuracy of the proposed system is at the centimeter level.

**Figure 14:**
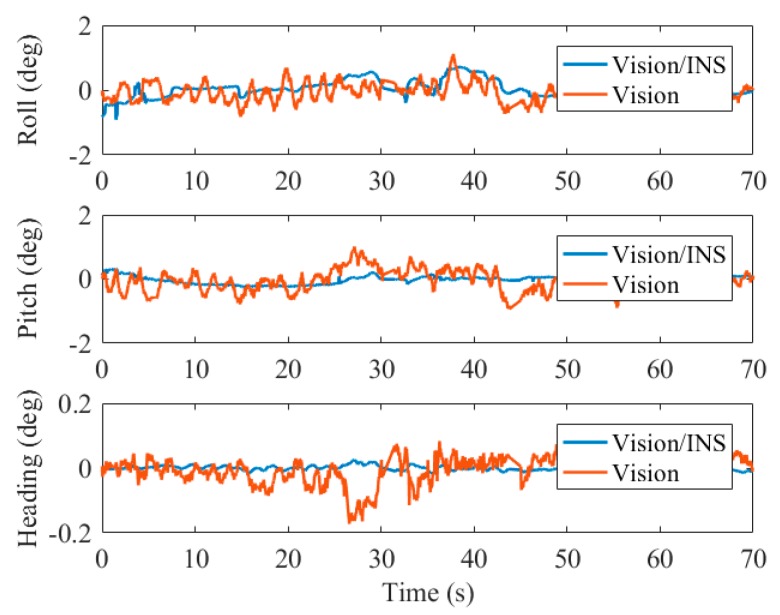
Comparison of roll and pitch errors by the visual solution and visual/inertial solution in test 04.

For the attitude errors, it can be observed from the statistical results in Table 5 that the roll angle error is 0.30° (1σ), the pitch angle error is 0.20° (1σ), and the heading angle error is 0.07° (1σ). Comparing to the pure visual solution, the roll and pitch angle accuracies have increased by 33% and 54% on average, respectively. Additionally, Figure 14 shows that the pitch angle and roll angle estimated by the integrated navigation are more stable and smooth than those of the visual solution. Due to the excellent sensibility to vehicle motion of the IMU, the horizontal angle vibration caused by the maneuver is alleviated by the fusion with INS.

**Figure 15:**
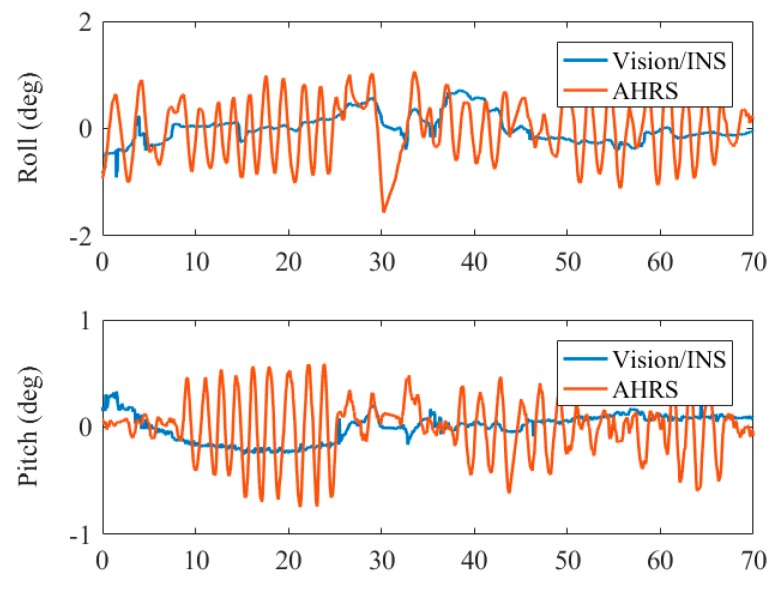
Comparison of roll and pitch errors by the attitude and heading reference system (AHRS) solution and visual/inertial solution.

The attitude and heading reference systems (AHRS) [43,44] is widely used for continuous attitude estimation for flight control of UAVs since it is independent of GNSS. It exploits the IMU for pitch and roll angle estimation and magnetometer augmentation for improving heading stability. Because there was no magnetometer in our test device, we used the IMU data for AHRS and compared the accuracy and stability of the horizontal angles. The Mahony AHRS algorithm [45] was performed in the test. Figure 15 illustrates the roll and pitch errors of the two systems in the fourth test.

It can be observed that the roll and pitch angle estimation of the proposed system is much more accurate and stable than that of the AHRS. The AHRS provides pitch and roll angles relative to the earth gravity vector, thus, the accuracy of horizontal angles suffers from the vehicle acceleration since it cannot be separated by IMU only. In the tests, we held the platform to move in circles, therefore, the direction-alternating acceleration resulted in the sinusoidal curve of the angles error of the AHRS. As for the horizontal angles estimated by the proposed system, the error of INS-derived position was related to the attitude error, hence the difference between the high-precision position obtained by marker detection and that of INS could correct the error of roll and pitch angles by the filter.

**Figure 16:**
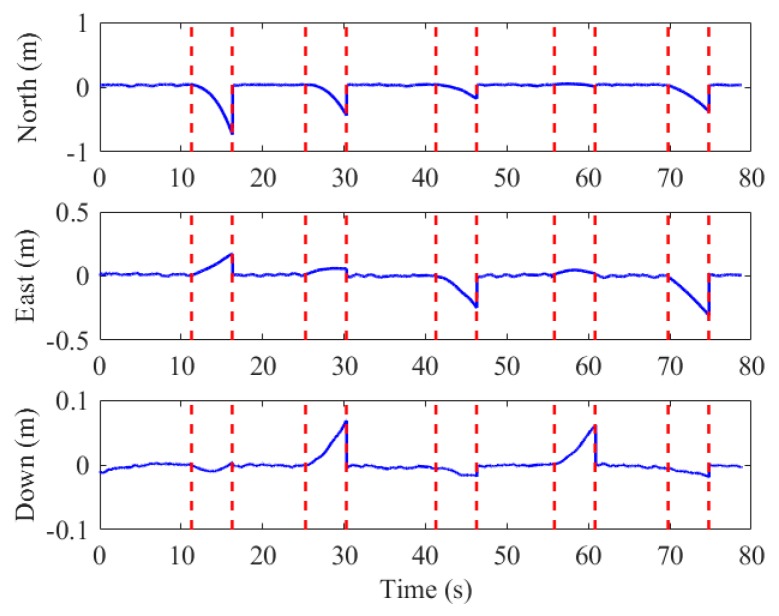
Position errors of the visual-inertial solution in the vision outage simulation test.

**Figure 17:**
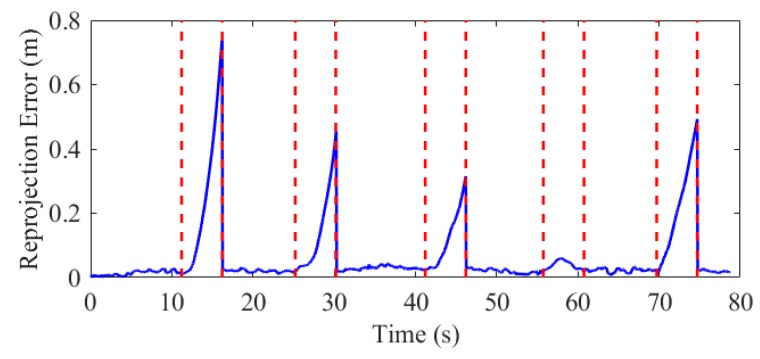
Reprojection error in the vision outage simulation test.

As aforementioned, INS can provide continuous positioning results when the external aiding source is short-term unavailable. To illustrate that our system is robust to failure in marker extraction, we artificially interrupted the visual correction in data processing and calculated the errors of the positioning results. We set five outages for each test, and each outage was set to 5 s. Figure 16 and Figure 17 are the position and reprojection errors of the simulation experiments in the fifth test, respectively.

The position error in the horizontal and vertical direction for the 25 outages in all the tests is 0.41 and 0.09 m (1σ), respectively, indicating that the proposed system can provide stable and reliable pose estimation when the marker is invalid occasionally. However, the navigation error of INS is accumulated with time as shown in Figure 17, hence long-term failure of marker detection will decay the performance.

**Figure 18:**
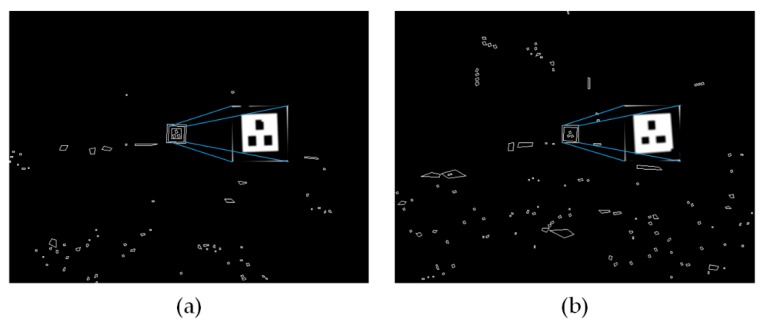
Edge extraction and marker detection results in challenging situations. (**a**) Detected marker at a distance of 8 m with no tilt; (**b**) detected marker at a distance of 8 m with tilt of 40 degrees.

To verify the robustness of the proposed system when the UAV is at a relative higher altitude or large-angle maneuver, we held the test platform 8 m away to capture the marker with no slant and with 40 degrees tilting, respectively. As the distance increases, the marker in the image becomes smaller and the field of view becomes larger, hence objects of different shapes around the marker came into view which caused the debris in the figures. Only the polygon containing correct grayscale information would be matched. Figure 18 presents the contours extraction and marker detection results in the two situations. The positioning errors (RMS) on vertical direction for these two tests are 0.12 and 0.23 m, respectively. The errors on horizontal direction are 0.21 and 0.30 m, respectively. It can be observed that the long distance and large tilt angle did not affect the marker detection. However, the positioning error increased since the marker was small on the image and the geometric condition of corner points was poor. Because the precision landing of the UAV depends primarily on the pose accuracy when it is close to the landing pad, this positioning results at such a distance are enough for the UAV to approach the target.

Finally, we record the time spent on each module of the system over 1000 trials on the Tegra K1 developer kit (NVIDIA, Santa Clara, CA, USA), including a 4-Plus-1 ARM Cortex-A15 CPU. The results are shown in Table 7.

It can be observed form the time consumption in Table 7 that the system runs efficiently. The most time-consuming part is marker detection, which requires a large number of pixel operations, while the other parts take relatively shorter times since they only need to process a small amount of low-dimension data. Overall, the average time consumption of the entire system is 32.26 ms, which is able to meet the real-time control requirements of UAVs.

## 4. Conclusions

In this paper, an artificial marker and MEMS IMU-based six-DoF pose estimation solution to meet the UAV landing requirements is proposed. The marker is specially designed for UAV landing due to its simplicity to generate and effectiveness to detect. An onboard monocular camera is used to recognize the marker and recover the absolute position and attitude of the UAV in the world frame. Then, this information is fused with IMU data by an EKF, thus our system still works in the case of visual failure, which is a fatal problem for previous systems. Improving upon the previous visual/inertial landing systems, the error components of the inertial sensor are modeled, and the horizontal angles are also estimated. Experiments show that the position accuracy is at centimeter level, the heading error is less than 0.1°, and the roll and pitch angle errors are less than 1° with the UAV at a height of about 1.4 m. The visual/inertial fused system improves the roll and pitch accuracy by 33% and 54%, respectively. Moreover, vision outage simulation tests show our system can provide relatively reliable position estimation (0.41 m drift in the horizontal direction and 0.09 m in the vertical direction in 5 s) when the maker occasionally occluded. Tests in challenging situations prove that the marker can be detected at a distance of 8 m and tilt of 40 degrees and the positioning accuracy is at decimeter level. The statistics of time consumption of the proposed system in an onboard computer show that it is able to meet the requirement of real-time control. The proposed system also applies to marker-based state estimation of indoor ground vehicles.

Future works will extend pose estimation to the entire route of UAV flight. Natural features are going to be used to estimate the UAV pose, when there is no marker in the field of vision. The marker can be used to correct the accumulated navigation error as long as it is detected.

## Figures and Tables

**Table 1:** Technical parameters of the micro-electromechanical system (MEMS) IMU and POS320.

Parameters	MEMS IMU (System under Test)	POS320 (Reference System)
Gyro bias stability (°/h)	200	0.5
Angle random work $\left({}^{\circ}/\sqrt{h}\right)$	0.24	0.05
Accelerometer bias stability $\left(m/s^{2}\right)$	0.01	0.00025
Velocity random work $\left(m/s/\sqrt{h}\right)$	3	0.1

**Table 2:** Statistical results of the position errors of the visual solution.

Statistics		01	02	03	04	05
North Error (m)	RMSE	0.007	0.006	0.008	0.006	0.006
	MAX	0.024	0.029	0.025	0.021	0.031
	MEAN	0.006	0.004	0.007	0.005	0.004
East Error (m)	RMSE	0.008	0.009	0.010	0.008	0.007
	MAX	0.025	0.025	0.024	0.034	0.019
	MEAN	0.007	0.007	0.008	0.007	0.006
Down Error (m)	RMSE	0.003	0.003	0.004	0.004	0.003
	MAX	0.013	0.010	0.013	0.012	0.014
	MEAN	0.003	0.003	0.003	0.003	0.002

RMSE: root mean square error; MAX: maximum.

**Table 3:** Statistical results of the attitude errors of the visual solution.

Statistics		01	02	03	04	05
Roll Error (°)	RMSE	0.45	0.53	0.54	0.38	0.34
	MAX	1.45	1.58	1.75	1.47	1.14
	MEAN	0.37	0.42	0.41	0.31	0.27
Pitch Error (°)	RMSE	0.37	0.49	0.55	0.39	0.35
	MAX	1.12	1.33	1.76	1.52	1.05
	MEAN	0.29	0.41	0.45	0.30	0.28
Yaw Error (°)	RMSE	0.05	0.08	0.09	0.05	0.05
	MAX	0.15	0.28	0.29	0.13	0.20
	MEAN	0.04	0.05	0.06	0.04	0.04

°: degrees.

**Table 4:** Statistical results of the position errors by the visual/inertial solution.

Statistics		01	02	03	04	05
North Error (m)	RMSE	0.007	0.006	0.009	0.007	0.006
	MAX	0.025	0.180	0.029	0.022	0.034
	MEAN	0.006	0.005	0.007	0.005	0.005
East Error (m)	RMSE	0.009	0.009	0.010	0.008	0.008
	MAX	0.033	0.031	0.025	0.034	0.023
	MEAN	0.007	0.007	0.008	0.007	0.006
Down Error (m)	RMSE	0.004	0.004	0.005	0.004	0.004
	MAX	0.013	0.010	0.015	0.010	0.012
	MEAN	0.003	0.003	0.003	0.004	0.003

**Table 5:** Statistical results of the attitude errors by the visual/inertial solution.

Statistics		01	02	03	04	05
Roll Error (°)	RMSE	0.08	0.44	0.34	0.21	0.43
	MAX	0.24	1.66	1.50	0.88	1.86
	MEAN	0.06	0.24	0.20	0.17	0.28
Pitch Error (°)	RMSE	0.17	0.14	0.24	0.27	0.17
	MAX	0.83	0.58	0.80	1.03	0.70
	MEAN	0.11	0.08	0.19	0.21	0.12
Yaw Error (°)	RMSE	0.06	0.13	0.07	0.05	0.03
	MAX	0.13	0.36	0.32	0.12	0.15
	MEAN	0.05	0.10	0.05	0.04	0.02

**Table 6:** Statistical results of the corner reprojection error.

Statistics	01	02	03	04	05
RMSE (m)	0.022	0.023	0.021	0.020	0.024
MAX (m)	0.042	0.049	0.049	0.033	0.044
MEAN (m)	0.020	0.020	0.018	0.019	0.023

**Table 7:** Running time statistics of different modules of the system.

Module	MAX (ms)	MEAN (ms)
Marker detection	36.15	25.21
Pose estimation by marker	9.01	1.71
Filtering update	1.21	0.25
Total	70.34	32.26
